# Importância do Exame Clínico na Avaliação dos Perfis Hemodinâmicos e sua Relação com Desfechos em Pacientes com Insuficiência Cardíaca Aguda

**DOI:** 10.36660/abc.20240308

**Published:** 2024-07-23

**Authors:** Suellen Rodrigues Rangel Siqueira, Vera Maria Cury Salemi

**Affiliations:** 1 Centro de Cardiologia do Hospital Sírio Libanês São Paulo SP Brasil Centro de Cardiologia do Hospital Sírio Libanês, São Paulo, SP – Brasil; 2 Instituto do Coração Hospital das Clínicas da FMUSP São Paulo SP Brasil Instituto do Coração (InCor), do Hospital das Clínicas da FMUSP, São Paulo, SP – Brasil

**Keywords:** Insuficiência Cardíaca/fisiopatologia, Mortalidade, Exame Médico, Hemodinâmica, Prognóstico

As doenças cardiovasculares são as principais causas de mortalidade no Brasil e no mundo e a insuficiência cardíaca (IC) é uma das causas mais prevalentes de morbi-mortalidade. Nos últimos três meses tivemos uma média mensal de 15.406 internações por IC no Brasil segundo dados coletados no DATASUS. É uma preocupação de saúde pública devido a sua alta morbidade, mortalidade e custo associado a internações frequentes. Assim, torna-se de fundamental importância que o diagnóstico seja rápido para que tratamento seja eficaz.^1^

O primeiro registro brasileiro de IC aguda incluiu 1263 pacientes de 51 centros brasileiros. O BREATH mostrou que as principais etiologias são a isquêmica, a hipertensiva, e a cardiomiopatia dilatada, seguidas de doenças valvares e de Chagas. A prevalência das etiologias é semelhante a registros internacionais, exceto pela doença de Chagas.^2^ A principal causa de rehospitalização foi a má aderência medicamentosa, além disso, o estudo mostrou elevada taxa de mortalidade intra-hospitalar. Uma doença tão prevalente e fatal necessita de um olhar criterioso e sistematizado para evitar falhas durante o tratamento.^3^

A fisiopatologia da IC aguda é complexa pois envolve um desarranjo hemodinâmico gerado por baixo débito cardíaco e aumento das pressões de enchimento. O baixo débito cardíaco leva a uma má perfusão periférica e aumento da pressão capilar pulmonar gera congestão.^4^ Em 1978, Forrester sugeriu uma avaliação sistemática do manejo da IC aguda baseada no seu perfil hemodinâmico, analisando a volemia e a perfusão do paciente tendo como parâmetros a pressão capilar pulmonar (PCP ≥ ou ≤18 mm Hg) e índice cardíaco (≥ 2,2 l/min/m²). O paciente podia ser dividido entre 4 categorias: seco e boa perfusão; congesto e boa perfusão; seco e perfusão ruim; congesto e perfusão ruim. Após a classificação de acordo com o perfil hemodinâmico, a escolha da terapia poderia ser direcionada com base em três classes de medicamentos que compõem o tratamento da IC aguda: diurético, vasodilatadores e inotrópicos. Nohria et al. realizaram uma adaptação dessa classificação usando apenas parâmetros clínicos para avaliação de congestão e perfusão. A congestão deve ser caracterizada por quadro clínico de ortopneia, estase de jugular, reflexo hepatojugular, ascite e edema de membros inferiores. A avaliação de perfusão deve ser baseada na pressão de pulso, pulso alternante, hipotensão sintomática, extremidades frias e confusão mental. A classificação teve uma nova nomenclatura: A- seco e quente, B- congesto e quente, C– congesto e frio, L- seco e frio. O interessante foi observar a diferença de sobrevida entre os diferentes perfis. Os pacientes classificados como perfil A tiveram melhor sobrevida e os paciente do grupo B e C eram os mais graves com maior mortalidade.^5–7^

O ESCAPE Trial reforçou a importância da avaliação clínica, hoje em dia não tão valorizada, quando mostra a não diferença de mortalidade entre os grupos clínico e com cateter de artéria pulmonar (CAP). O CAP tem seu lugar nos pacientes mais críticos com IC avançada e necessidade de inotrópico. Portanto, a avaliação clínica do perfil hemodinâmico do paciente é fundamental para um tratamento direcionado e precoce, evitando assim, a deterioração do paciente após internação hospitalar.^8^

No artigo Associação entre o Perfil Hemodinâmico da Insuficiência Cardíaca à Admissão Hospitalar e Mortalidade - Programa Boas Práticas Clínicas em Cardiologia,^9^ o perfil clínico dos pacientes com IC aguda no Brasil incluiu 1.978 pacientes, idade média de 60 anos, a maioria dos pacientes do gênero masculino, de baixa escolaridade e baixa renda, com fração de ejeção média do ventrículo esquerdo de 39,8%, mostrou predominância no perfil B, semelhante aos resultados mundiais, e reinternação maior nos pacientes congestos (frio ou quente). A mortalidade geral da IC aguda na literatura varia entre 1,8 e 36%, e essa variação está associada aos diferentes perfis clínicos e hemodinâmicos dos pacientes. O presente estudo realizado em nosso país mostra que a mortalidade foi maior nos perfis hemodinâmicos frios, além de um maior tempo de internação nos hospitais do Brasil comparado com coortes internacionais e uma maior mortalidade comparando a população norte americana e europeia.^10^ Apesar desse estudo ter sido realizado na grande maioria em hospitais terciários, talvez esses dados tenham sido influenciados pela população com baixa renda, refletindo uma população mais grave e com acesso precoce mais limitado ao sistema de saúde, reforçando ainda mais a necessidade de políticas públicas para atenção básica. Esses dados devem servir de alerta e mostrar a importância da realização do diagnóstico precoce e tratamento imediato de pacientes com IC aguda. A avaliação clínica pelo perfil hemodinâmico é um método rápido de estratificação da gravidade do paciente, que deve ser usado à beira do leito e sem custo, mostrando os níveis de gravidade, para ter a possibilidade de aplicação rápida de protocolos institucionais de tratamento e de seguimento do paciente após a alta hospitalar.
