# Supplementary material for: Importância do Exame Clínico na Avaliação dos Perfis Hemodinâmicos e sua Relação com Desfechos em Pacientes com Insuficiência Cardíaca Aguda
Source: Arq Bras Cardiol. 2024 Jul 23;121(5):e20240308. [Article in Portuguese] doi: 10.36660/abc.20240308 (PMC11364442; doi:10.36660/abc.20240308)
Supplement: Translation

# Translation: Importance of Clinical Examination in the Assessment of Hemodynamic Profiles and Their Relationship with Outcomes in Patients with Acute Heart Failure

**DOI:** 10.36660/abc.20240308i

**Authors:** Suellen Rodrigues Rangel Siqueira, Vera Maria Cury Salemi

**Affiliations:** 1 São Paulo SP Brazil Centro de Cardiologia do Hospital Sírio Libanês, São Paulo, SP – Brazil; 2 São Paulo SP Brazil Instituto do Coração (InCor), do Hospital das Clínicas da FMUSP, São Paulo, SP – Brazil

**Keywords:** Heart Failure/physiopathology, Mortality, Medical Examination, Hemodynamic, Prognosis

Cardiovascular diseases are the main causes of mortality in Brazil and worldwide and heart failure (HF) is one of the most prevalent causes of morbidity and mortality. In the last three months, we had a monthly average of 15,406 hospitalizations for HF in Brazil according to data collected in DATASUS. It is a public health concern due to its high morbidity, mortality, and cost associated with frequent hospitalizations. Therefore, it is of fundamental importance that the diagnosis is rapid so that treatment is effective.^1^

The first Brazilian registry of acute HF included 1263 patients from 51 Brazilian centers. BREATH showed that the main etiologies are ischemic, hypertensive, and dilated cardiomyopathy, followed by valvular and Chagas disease. The prevalence of etiologies is similar to international records, except for Chagas disease.^2^ The main cause of rehospitalization was poor medication adherence, in addition, the study showed a high in-hospital mortality rate. Such a prevalent and fatal disease requires a careful and systematic approach to avoid failures during treatment.^3^

The pathophysiology of acute HF is complex as it involves a hemodynamic disorder generated by low cardiac output and increased filling pressures. Low cardiac output leads to poor peripheral perfusion and increased pulmonary capillary pressure generates congestion.^4^ In 1978, Forrester suggested a systematic assessment of the management of acute HF based on its hemodynamic profile, analyzing the patient's blood volume and perfusion using the parameters pulmonary capillary pressure (PCP ≥ or ≤18 mm Hg) and cardiac index (≥ 2.2 l/min/m²). The patient could be divided into 4 categories: dry and good perfusion; congestion and good perfusion; dryness and poor perfusion; and congestion and poor perfusion. After classification according to the hemodynamic profile, the choice of therapy could be directed based on three classes of medications that make up the treatment of acute HF: diuretics, vasodilators, and inotropes. Nohria et al. performed an adaptation of this classification using only clinical parameters to assess congestion and perfusion. Congestion must be characterized by a clinical picture of orthopnea, jugular stasis, hepatojugular reflex, ascites, and edema of the lower limbs. Perfusion assessment should be based on pulse pressure, alternating pulse, symptomatic hypotension, cold extremities, and mental confusion. The classification had a new nomenclature: A- dry and hot, B- congested and hot, C- congested and cold, L- dry and cold. The interesting thing was to observe the difference in survival between the different profiles. Patients classified as profile A had better survival and patients in groups B and C were the most severely ill with higher mortality.^5–7^

The ESCAPE Trial reinforced the importance of clinical evaluation, which nowadays is not so valued when it showed no difference in mortality between the clinical and pulmonary artery catheter (PAC) groups. PAC has its place in the most critical patients with advanced HF and need of inotropes. Therefore, clinical assessment of the patient's hemodynamic profile is essential for targeted and early treatment, thus avoiding patient deterioration after hospital admission.^8^

In the article Association between Hemodynamic Profile at Hospital Admission and Mortality in Acute Heart Failure Patients Included in the Best Practice in Cardiology Program in Brazil,^9^ the clinical profile of patients with acute HF in Brazil included 1,978 patients, a mean age of 60 years, the majority of patients male, with low education and low income, with a mean left ventricular ejection fraction of 39.8%, showed a predominance in profile B, similar to global results, and higher readmissions in congested patients (cold or hot). The general mortality of acute HF in the literature varies between 1.8 and 36%, and this variation is associated with the different clinical and hemodynamic profiles of the patients. The present study carried out in our country shows that mortality was higher in cold hemodynamic profiles, in addition to a longer length of stay in Brazilian hospitals compared to international cohorts and a higher mortality comparing the North American and European populations.^10^ Although this study has the vast majority of cases carried out in tertiary hospitals, perhaps these data were influenced by the low-income population, reflecting a more severe population with more limited early access to the health system, further reinforcing the need for public policies for basic care. These data should serve as a warning and show the importance of early diagnosis and immediate treatment of patients with acute HF. Clinical assessment using the hemodynamic profile is a quick method of stratifying the patient's severity, which should be used at the bedside and at no cost, showing severity levels, to have the possibility of rapid application of institutional treatment and follow-up protocols. of the patient after hospital discharge.
